# The effect of HD-tDCS on brain oscillations and frontal synchronicity during resting-state EEG in violent offenders with a substance dependence

**DOI:** 10.1016/j.ijchp.2023.100374

**Published:** 2023-02-21

**Authors:** Carmen S. Sergiou, Elisa Tatti, Sara M. Romanella, Emiliano Santarnecchi, Alix D. Weidema, Eric G.C Rassin, Ingmar H.A. Franken, Josanne D.M. van Dongen

**Affiliations:** a Department of Psychology, Education and Child Studies, Erasmus University Rotterdam, Rotterdam, the Netherlands; b Berenson-Allen Center for Non-Invasive Brain Stimulation, Beth Israel Medical Center, Harvard Medical School, Boston, MA, USA; c City College of New York (CUNY) School of Medicine, New York, NY, USA

**Keywords:** High definition-transcranial direct current stimulation, Resting-state electroencephalography, Violent offenders, brain oscillations, frontal brain regions

## Abstract

Violence is a major problem in our society and therefore research into the neural underpinnings of aggression has grown exponentially. Although in the past decade the biological underpinnings of aggressive behavior have been examined, research on neural oscillations in violent offenders during resting-state electroencephalography (rsEEG) remains scarce. In this study we aimed to investigate the effect of high-definition transcranial direct current stimulation (HD-tDCS) on frontal theta, alpha and beta frequency power, asymmetrical frontal activity, and frontal synchronicity in violent offenders.

Fifty male violent forensic patients diagnosed with a substance dependence were included in a double-blind sham-controlled randomized study. The patients received 20 minutes of HD-tDCS two times a day on five consecutive days. Before and after the intervention, the patients underwent a rsEEG task.

Results showed no effect of HD-tDCS on the power in the different frequency bands. Also, no increase in asymmetrical activity was found. However, we found increased synchronicity in frontal regions in the alpha and beta frequency bands indicating enhanced connectivity in frontal brain regions as a result of the HD-tDCS-intervention.

This study has enhanced our understanding of the neural underpinnings of aggression and violence, pointing to the importance of alpha and beta frequency bands and their connectivity in frontal brain regions. Although future studies should further investigate the complex neural underpinnings of aggression in different populations and using whole-brain connectivity, it can be suggested with caution, that HD-tDCS could be an innovative method to regain frontal synchronicity in neurorehabilitation.

## Introduction

Aggression is a major problem in our society (Grochowska and Kossowska 2012; Mooney and Daffern 2011, 2015), and consequently, research into the neural underpinnings of aggression has grown exponentially. Studies on aggressive behavior have previously shown that dysfunctions within the temporal and frontal lobes are associated with violence (Convit, 1991; Gatzke-Kopp et al., 2001; Tancredi & Volkow, 1988; Volkow & Tancredi, 1987; Volavka, 1990), and that these dysfunctions predict later criminal behavior (Lewis et al., 1985; Raine et al., 1990). In addition, there are studies that have shown that the prefrontal cortex (PFC), and specially the ventromedial prefrontal cortex (vmPFC), is the core brain region that is involved in regulating aggressive behavior (Gizewski et al., 2013; Fanning et al., 2017, Kose et al., 2015; Marxen et al., 2016; Preller et al., 2014; Sergiou et al., 2020a,b; Yang and Raine, 2009), antisocial behavior, especially aggression and impulsivity and that this is present in abusers of alcohol and cocaine (Anderson et al., 1999; Blair, 2004; Calzada-Reyes et al., 2013, 2017; Krämer et al., 2009; Raine, 1993; Raine et al. 2000). For instance, a review study by Bufkin and Lutrell (2005) described several neuroimaging studies of aggressive, violent or antisocial individuals demonstrating that diminished functioning of the prefrontal cortex was associated to their aggressive behavior and deficits in the vmPFC could lead to increased violent behavior (Blair, 2001, 2005, 2006).

One way for studying dysfunctions in brain structures is using resting-state electroencephalography (rsEEG). In the past decades, rsEEG has received increased attention due to its unique advantages in investigating brain connectivity and characterizing changes in brain functions among different psychiatric disorders. Resting-state is the state in which an individual is awake, but not engaged in a specific task allowing to reflect the intrinsic activity of the brain (Deco et al., 2011; Greicius et al., 2003). Temporal dynamic patterns of the brain activity in resting state measured by EEG are suggested to change more rapidly than can be detected using techniques such as functional Magnetic Resonance Imaging (fMRI; Baker et al., 2014; Deco & Corbetta, 2011; Siegel et al., 2012). In addition, EEG is a favorable technique to measure temporal dynamics because it is more suited to investigate the mechanisms underlying neural communication as EEG can directly measure the electrophysiological activity (Ganzetti & Mantini, 2013; Pfurtscheller & Lopes da Silva, 1999). In that matter it can demonstrate the organization of spontaneous brain oscillations and interactions between different networks with high temporal resolution (Liu et al., 2018; Siegel et al., 2012). rsEEG enables the investigation of electrical fluctuations in neural signals, allowing brainwave patterns during rest and thought processing to be detected (Jorge et al., 2014). (These neural oscillations can be divided into different frequency bands, which are ranging from the delta wave (δ; 0.3–3.5 Hz), theta wave (θ; 4–7.5 Hz), alpha wave (α; 8–13 Hz), beta wave (β; 14–30 Hz) to the gamma wave (γ; >30 Hz), which are linked to different emotional states and cognition (Di et al., 2018). Various formations of neural synchrony in oscillations over the different frequency bands are proposed to be the major structure of neural integration (Munia & Aviyente, 2019). Studies investigating the electrophysiological estimation of neural activity suggest that these different frequency bands can be linked to distinct central processes (Da Silva, 2013) and that the oscillations create a synchronization over various brain regions to endorse cognitive processing (Engel et al., 2013; Hall et al., 2014; Schölvinck et al., 2013; Stam, 2014; Tewarie et al., 2015).

With regard to criminal behavior and violence, previous research has shown decreased arousal in the frontal brain regions (Decety et al., 2015); demonstrating enhanced slow-wave activity of the delta (0.5–3.5 Hz) and theta band (3.5–7.5 Hz) in the brain of antisocial individuals, and decreased alpha activity in criminals (Ellingson, 1954; Mednick et al., 1981; Raine et al.,1990). Common findings in these studies investigating EEG of antisocial and violent individuals show that this excessive slow-wave activity is related to aggressive tendencies within these individuals (Hill & Waterson, 1942; Scarpa et al., 1997; Brower & Price, 2001; Moya-Albiol, 2004).

Furthermore, besides the higher power of activity in slow waves, the reduced power in the alpha frequency band (8–12 Hz) also seems one of the more robust findings in individuals with antisocial and behavioral disorders (Calzada-Reyes et al, 2013; Convit et al., 1991; Fishbein et al., 1989; Forssmann & Frey, 1953; Lindberg et al., 2005; Keune et al., 2012). Alpha frequency has been linked to inhibition (Klimesch et al., 2007; Weisz et al., 2007) and is proposed to represent communication between different brain regions on a long range (von Stein et al., 2000). Given the impaired neural activity of violent offenders in the alpha frequency band, it is plausible that impaired brain communication reflects one of the neuroanatomical key factors to antisocial and violent individuals (Motzkin et al., 2011; Yang & Raine, 2009). In sum, these studies indicate that violent offenders show abnormalities in frontal regions, including enhanced power in the theta frequency band and decreased alpha power and impaired connectivity.

In addition to dysfunctions in the power in different frequency bands, increasing evidence suggests asymmetrical involvement of the frontal hemispheres in aggressive behavior (for review see Harmon-Jones, 2003). Particularly, the left hemisphere has been found dominant in aggressive behavior (Carvor & Harmon-Jones, 2009; Peterson et al., 2008; Schutter et al., 2008), and studies link increasing activity with higher levels of experienced anger and aggressive behavior (Peterson et al., 2008). Furthermore, the studies of Hofman and Schutter (2009, 2012) have demonstrated that asymmetrical activity in the beta frequency band specifically is found to be related to the development and expression of aggressive behavior (Hofman & Schutter, 2012) and in trait aggression in healthy individuals. This frontal asymmetry in aggression might be understood in terms of increased approach-related motivational tendencies (i.e. the left hemisphere) and decreased levels of avoidance-related motivational tendencies (i.e. the right hemisphere; Harmon-Jones and Allen, 1998; Harmon-Jones, 2007; Schutter et al., 2008).

Furthermore, an interesting technique to examine the synchronicity and communication between frontal regions is to measure the intercorrelation in phase between electrodes. Synchronization can be described as a type of connectivity measure and refers to a process in which linear or nonlinear oscillatory components reflect a property in activity over time, demonstrating a collective behavior (Boccaletti et al., 2018). In this way the interaction between brain areas can be detected (Sengupta et al., 2014). Several indices can be used to determine the intercorrelation in phase between electrodes, one of these methods is the Phase Locking Value (PLV; Lachaux et al., 1999), which determines the coherence in phase between electrodes. The PLV is found to have the capability to determine phase coherence independent of the spectral power of the recorded signals and to be a robust measure in case of noisy signals (Cohen, 2015). Finally, the PLV is a more sensitive measure of synchronicity compared to other synchronicity measures when an investigation is driven by a hypothesis (Cohen, 2015) and best fitted to use when rsEEG data is involved.

A compelling way of investigating the dynamics of brain oscillations in an experimental way, is to combine EEG with non- invasive brain stimulation (NIBS). Using NIBS, measuring changes in brain activity and capturing individual variability or disorder specific alterations is possible (Benussi et al., 2020; Massimini et al., 2012; Voineskos et al., 2010). A promising NIBS device is High-Definition transcranial Direct Current Stimulation (HD-tDCS), which uses a high-definition technique targeting brain regions with higher focality (DaSilva et al., 2015), and in this way, modulates deeper lying brain regions in the most optimal way (Edwards et al., 2013; Hess, 2013; Nakamura-Palacios et al., 2016; Manuel et al., 2014; Tanaka et al., 2013).

Regarding tDCS in studying aggression, a recent study of Sergiou et al. (2022) multisession HD-tDCS targeting the vmPFC resulted in a decrease of aggressive responses on a laboratory aggression task and questionnaire in violent offenders. Another study (Molero-Chamizo et al., 2019) used one-session tDCS to reduce self-report aggression in a prison sample. In addition, other studies reduced aggressive tendencies (Choy et al., 2018; Dambacher et al., 2015; Gilam 2018; Ling et al., 2020) and unprovoked aggression (Riva et al., 2017) using traditional tDCS in healthy individuals. In the same vein, Although brain stimulation was traditionally used to target single brain regions of interest to modulate brain activity and neurological deficit, recently multiple studies demonstrate that the effect of the neuromodulation device is not limited to the stimulated region underlying the electrodes, but it can resonate into cortical networks and changes in functional connectivity (Di Lazzaro, 2004; Massimini et al., 2005; Siebner et al., 2001). As the neural signal reflects fluctuation of potentials resulting from postsynaptic potentials of cortical neurons which can be measured with EEG and therefore the brain areas directly or indirectly affected by tDCS can be investigated (Mangia et al., 2014). Multiple studies have demonstrated that neuromodulation techniques can influence synchronicity, both during task performance as in resting state (Alon et al., 2011; Chib et al., 2013; Ding et al., 2014; Grefkens et al., 2010; Keeser et al., 2011a; Meinzer et al. 2012, 2013; Peña-Gómez et al., 2012; Park et al., 2013; Polanía et al., 2011a, 2012; Stagg et al., 2013; Vanneste & de Ridder, 2011; Weber et al., 2014), others found increased functional connectivity patterns (Antal et al., 2004a, 2004b, Ardolino et al., 2005; Notturno et al., 2013; Polanía et al., 2012) and enhanced power in alpha and beta after anodal stimulation of the DLPFC (Zaehle et al., 2011a, 2011b) in healthy individuals. In the same vein, studies (Keeser et al., 2011a; Hampstead et al., 2013) demonstrated that tDCS increased the interaction between inter- and intra-cerebral cortexes and an enhancement of the coherence of the cerebral rhythm and functional organization at rest (Polanía et al., 2012). Investigating the synchronicity may thus be very important to picture deficits in the brain in different neurological or psychiatric disorders and in aggressive behavior (Fox, 2018; Fox et al., 2012; Sheffield & Barch, 2016). Since all the above-mentioned studies used a sample of healthy individuals, and not in relation with aggression, it would be interesting to add to the current literature by investigate alterations in the frontal region of violent offenders in resting-state to determine the effect of HD-tDCS in altering activity.

Although in the past decade the neurobiological underpinnings of antisocial personality, violent behavior and crime have been received increased attention (i.e., Convit, 1991; Gatzke-Kopp et al., 2001; Tancredi & Volkow, 1988; Volkow & Tancredi, 1987; Volavka, 1990), investigating associated deficits in neural oscillations during rsEEG in violent offenders remains scarce (i.e. Calzada-Reyes et al, 2013; Convit et al., 1991; Fishbein et al., 1989). Furthermore, neuromodulation technique as a tool to modulate brain oscillations in a violent offender sample including its effect on frequency power, asymmetrical activity and phase-synchronization is not previously investigated yet..In the current study we aimed to add to the scarce HD-tDCS-rsEEG research in violent offenders by investigating whether HD-tDCS could modulate brain oscillations in a violent offender sample. We specifically focused on frontal brain oscillations because functioning in the frontal brain region is found to be directly associated with violent behavior (Gizewski et al., 2013; Fanning et al., 2017, Kose et al., 2015; Marxen et al., 2016; Preller et al., 2014; Sergiou et al., 2020a,b; Yang and Raine, 2009). In addition, previous studies investigating asymmetrical activity also focus on the frontal activity of the brain in relation with aggression (i.e., see review Harmon-Jones, 2003). In the current study violent behaviors were studied in the context of addiction and/or alcohol use disorder, and specifically occurred during the abstinent phase of the addiction cycle.

The aim of this paper was threefold in to unravel the electrophysiological properties of aggression by analyzing the rsEEG of violent offenders using three different brain oscillation methods (spectral power, asymmetrical activity and synchronicity).

(i) first we examined whether HD-tDCS targeting the vmPFC could modulate power within the proposed frequency bands (theta, alpha, beta) over our electrodes of interest (i.e., in the prefrontal cortex). Based on previous literature we hypothesized an increase in activity the alpha frequency band (8–12 Hz), a decrease in the activity of the slow frequencies in theta frequency bands (4–7 Hz) in the violent offender sample after the HD-tDCS intervention.

Second, (ii) since asymmetrical activity in the frontal alpha and beta frequency bands has been linked to aggressive behavior (i.e., Hortensius & Schutter, 2012; Schutter & Hortensius, 2009), we aimed at investigating whether a change in the asymmetry of alpha and beta from could be established from pre to poststimulation. We expected an increase for theright sided activity in both frequency bands. Last, (iii) this study examined frontal synchronicity by determining the phase-correlation or phase-locking value (PLV) between each possible pair of the eight frontal electrodes and compared these from pre to posttest to see whether the HD-tDCS intervention affected the strengthening of synchronization between the electrodes in the specific frequency bands. We hypothesized that there would be in an increase in the PLV in the alpha and beta frequency bands.

## Materials and methods

### Participants

Data were collected as part of a study examining the effects of HD-tDCS in increasing empathy and reducing violent behavior in forensic patients with a substance dependence (see Sergiou et al., 2020a; Sergiou et al., 2022). A total of 50 male participants (mean age = 37.40 years, *SD* = 9.19 years, range: 22–62 years) were recruited from two departments of the division for forensic mental healthcare and addiction of Antes in Poortugaal, the Netherlands (see Table 1 for an overview). Twenty-one participants were recruited at the Forensic Addiction Clinic (FVK) and 29 from the Department of Forensic Care (AFZ). Inclusion criteria were: age 18–60, a good understanding of the Dutch language, diagnosed with an alcohol and/or cocaine substance use disorder (SUD) according to The Diagnostic and Statistical Manual of Mental Disorders (DSM–5; American Psychiatric Association, 2013), patients had to be abstinent and had to be sentenced for a violent offense as determined by one of the most used mandatory risk assessment tools in the Netherlands (HKT-R; Spreen et al., 2014). Exclusion criteria included: major neurological conditions, or major mental disorders, taking antipsychotics or other confounding medication (See supplementary materials S1 for CONSORT). All participants gave written consent to participate in the study. The study was conducted in accordance with the ethical standard of the Declaration of Helsinki (General Assembly of the World Medical Association, 2014) and was approved by the Medical and Ethical Review Board of the Erasmus Medical Center Rotterdam.

**Table 1:** Demographic characteristics.

	tDCS group		Sham group	
	*n*	*%*	*n*	*%*
Caucasian	25	100	23	92
Non- Caucasian	0	–	2	8
Primary education	9	36	8	32
High School	7	28	6	24
Secondary education (VET)	9	36	11	44
DSM-5 Axis I	7	28	10	40
DSM-5 Axis II	8	32	10	40
Mono substance use	8	32	9	36
Poly substance use	17	68	16	64

*Note. Characteristics are displayed in percentage of participants per group, N = 50 (*n* = 25 for each condition). Participants were on average 36.4 years old (SD = 8.88) in the tDCS group and on average 38.4 years in the sham group (SD = 9.56), and participant age did not differ by condition. VET= Vocational Education and Training, DSM-5= Diagnostic Symptom Manual fifth edition.

### Methods

#### High-Definition transcranial direct current stimulation

HD-tDCS was administered with the CE-certified Neuroelectrics Starstim8 (Neuroelectrics Barcelona, SLU; 2011), and the protocol was based on the evidence-based guidelines of LeFaucheur et al. (2017). Before the commencement of the study, the HD-tDCS montage optimization was based on the current flow modeling of the NIC-software of the tDCS Starstim8 system (Neuroelectrics Barcelona, SLU; 2011), see Sergiou et al. (2020a, 2022) for further details. In addition, the induced E-field of the HD-tDCS montage was computed in SimNibs v3.2 (Thielscher et al., 2015).

**Fig. 1:**
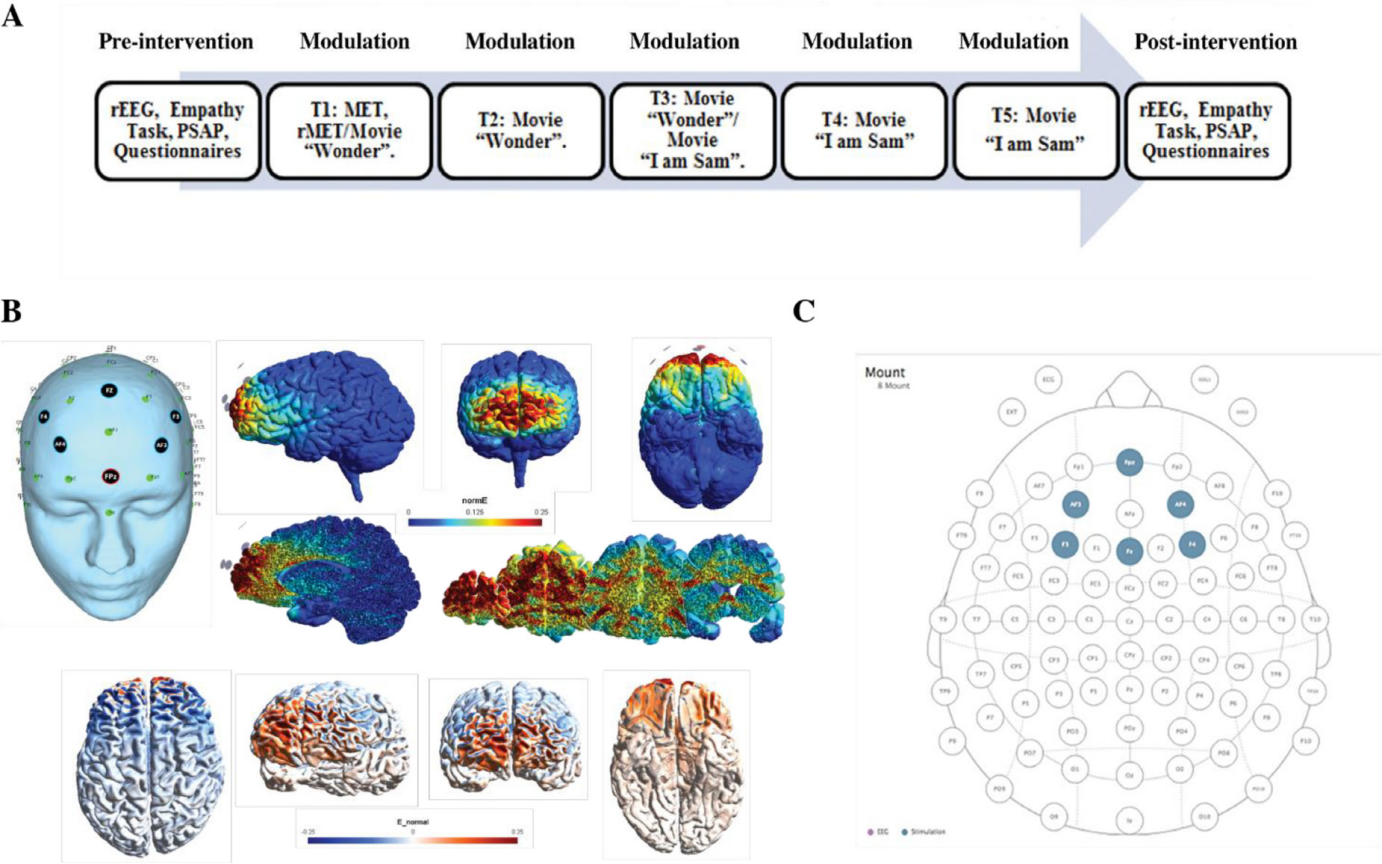
(A) Placement of the electrodes at 32 standard 10–20 electroencephalography system on the scalp with anodal high-definition transcranial direct current stimulation (HD-tDCS) with 6 3 1-cm radius (p cm^2^) electrodes over the Fpz (2 mA) and cathodal tDCS over AF3, AF4, F3, F4, and Fz (20.4 mA each). (B) Different views and slices of the map of electrical field induced by HD-tDCS montage as expressed in normE (V/m). This measure allows us to see the intensity of the stimulation independently by the polarity. (C) Views of the map of the electrical field expressed in normalE (E_normal) (V/m) showing the polarity (anodal/cathodal) of the stimulation.

The currents were transmitted through six circular Ag/AgCl PiStim “High Definition” electrodes (1 cm radius) that were applied with conductive gel. For the active condition the HD-tDCS device was programmed for stimulation targeting the vmPFC, with 2 mA HD-tDCS during 20 minutes. The sham condition followed the exact same procedure, only with 30 seconds ramping up and down of the HD-tDCS currents at the beginning and end of the protocol, based on earlier research indicating this method being effective for blinding (Gandiga et al., 2006). The anodal electrode was placed on the Fpz location, and the five return or cathodal electrodes were placed on AF3, AF4, F3, Fz and F4 (see Sergiou et al., 2022 for electrical field modeling and biophysical modeling). In Fig. 1 an overview of the HD-tDCS montage is presented.

#### Resting-state EEG (rsEEG) task

To assess the brain state in resting condition using rsEEG, the patients underwent a rsEEG task with eyes open condition (EO) and eyes closed (EC) condition oscillated every minute, for four consecutive minutes.

Data were collected when participants were sat comfortably with their eyes fixated on the screen. The recording lasted for about four minutes. (see Sergiou et al., 2020a; Sergiou et al., 2022). Since the cue for switching between the conditions of the EO and EC conditions was based on the sound of a bird (2130 ms) per trial, this was removed before further analysis, since this can evoke an auditory response.

#### EEG recording and preprocessing

Activity of the EEG was recorded using a mobile version of the BrainProducts (Live Amp, 2018) Active-Two System amplifier (Amsterdam, the Netherlands). Resting-state was recorded using a 4 min eyes open-eyes closed-eyes open-eyes closed recording session. Thirty- two electrodes have been placed on the scalp of each participant following the international 10- 20 EEG system. Two other additional electrodes were placed vertically above and beneath the left eye (electro-oculogram; EOG), the electrodes for the left and right mastoid placement were incorporated in the EEG cap.

The recording electrodes were positioned according to the extended 10–20 system and digitized at a sampling rate of 500 Hz. The impedance for all electrodes was maintained below 10 KΩ. The ground electrode was placed at the Pz position in both systems. Preprocessing was conducted in the open-source EEGLAB toolbox (Delorme and Makeig 2004) and custom MATLAB 2018b scripts (the Mathworks, Inc.).

The preprocessing pipeline included 1–70 Hz bandpass filtering, a notch filter at 50 Hz. Epochs of two seconds were created, resulting in four segments per participant (pre and post, EO and EC). Data were excluded based on data segments containing artifacts (EEGLAB, personalized pipeline) and the use of independent component analysis (ICA) removal of eye movement and muscle artifacts. If any of the two data sets (pre and post) of EO and EC, recorded from a given participant needed to be removed due to excessive artifacts or missing signal (i.e., if the number of remaining trials was less than 50%), that data was excluded from the analysis. Similarly, data from participants were removed if the signal from their resting- state recording that remained after cleaning had a total length of less than 240 s. The signals were re-referenced to average reference at the end of the script. Ultimately, we included only the EC condition for further analysis, since the EO condition was not of good quality and we want to examine this in a following study. In addition, studies (i.e., Barry et al., 2007) recommend using eyes-closed resting EEG as a baseline for all studies which do not use visual stimuli in their task, indicating that EC is a more valid way of measuring resting-state in our study.

#### Spectral power analysis

The power of three EEG bands (theta, 4–7 Hz; alpha, 8–13 Hz; and beta (14–30), was calculated for each electrode individually (averaged over trials) for each window of spectral power. The frequency-domain analysis was performed using the Darbeliai EEGLAB extension (Baranauskas, 2008), which makes use of a Fast Fourier Transform (FFT) algorithm to extract the absolute (μV^2^/Hz) power density, the relative (%) power density and the mean frequency (Hz) within each of the three frequency bands. The absolute power (AP) can be described as the integral of all of the power values in the frequency range of that band. Relative power (RP) is the absolute power calculated over the four frequency bands together (1–32 Hz). Mean (total) frequency (Hz) was also derived from the entire analyzed spectrum (1– 32 Hz). Mean frequency was calculated over the following electrodes for the theta, alpha and beta frequency band: F3, F4, F7, F8, Fz, Fp1, FC1 and FC2.

#### Asymmetrical frontal activity

Measures of inter-hemispheric (absolute) power asymmetry for each band were computed for three homologous sites (F3-F4, F7-F8, FC1-FC2) and mean spectral power for left vs right frontal activity was measured by averaging the four corresponding electrodes (see Schutter & Knyazev, 2012). Accordingly, the asymmetrical power distribution was calculated by *powerasymmetry = (power^right^ – power^left^) / (power^right^ + power^left^)* (Schutter et al., 2008) meaning that a greater value or increase in asymmetrical activity is right over left cortical activity. In this way there can be controlled for individual differences in skull thickness, skull- to-cortex distance and orientation of the underlying cortical tissue (Herbsman et al., 2009).

#### EEG synchronicity analysis

To examine the synchronicity between the frontal electrodes from pre to posttest, we used the Phase Locking Value (PLV), to calculate the phase symmetry between couples of electrodes (Fz, Fp1, F3, F4, F7, F8, FC1, FC2). We calculated this separately for the theta (4–7 Hz), alpha (8–13 Hz) and beta (14–30 Hz) frequency band and the two measurement moments (pretest vs. posttest). The PLV was calculated using a customized script in Matlab. Phase locking between selected electrode pairs of recording sites was calculated based on the PLV-method described in (Lachaux et al., 1999).

We extracted EEG brain synchronicity over 26 numbers of couples of electrodes in three EEG frequency bands (theta, alpha, beta) using a sliding windows approach. We chose to look at data for 1.998 sec continuously. For each frequency band, we then computed the Phase-Locking Value (PLV), a phase synchronization measure that corresponds to the mean phase difference between signals from two electrodes. This allowed us to investigate the phase synchronization between two narrow-band signals. To do so we use the Hilbert transform:

We then computed the relative phase between two signals:

The instantaneous PLV is computed with the formula:

We created a matrix of 26 PLV correlations of chosen electrodes in the prefrontal area (Fz-F3, Fz-F4, Fz-F7, Fz-F8, Fz-FP1, Fz-FC1, Fz-FC2, FP1-F3, FP1-F4, FP1-F7, FP1-F8, FP1-FC1, FP1-FC2, F3-F4, F3-F7, F3-F8, F3-FC1, F3-FC2, F4-F7, F4-F8, F4-FC1, F4-FC2, F7-FC1, F7-FC2, F8-FC1, F8-FC2). The PLV indicates the inter-trial variability of the phase association between pairs of brain regions as a function of time. Values closer to 1 indicate strong synchronicity (i.e., phase-locking) between two regions within the indicated time window, whereas values closer to 0 indicate phase variation between two electrodes, and thus represent a weak synchronicity between regions (Spooner et al., 2020).

**Fig. 2:**
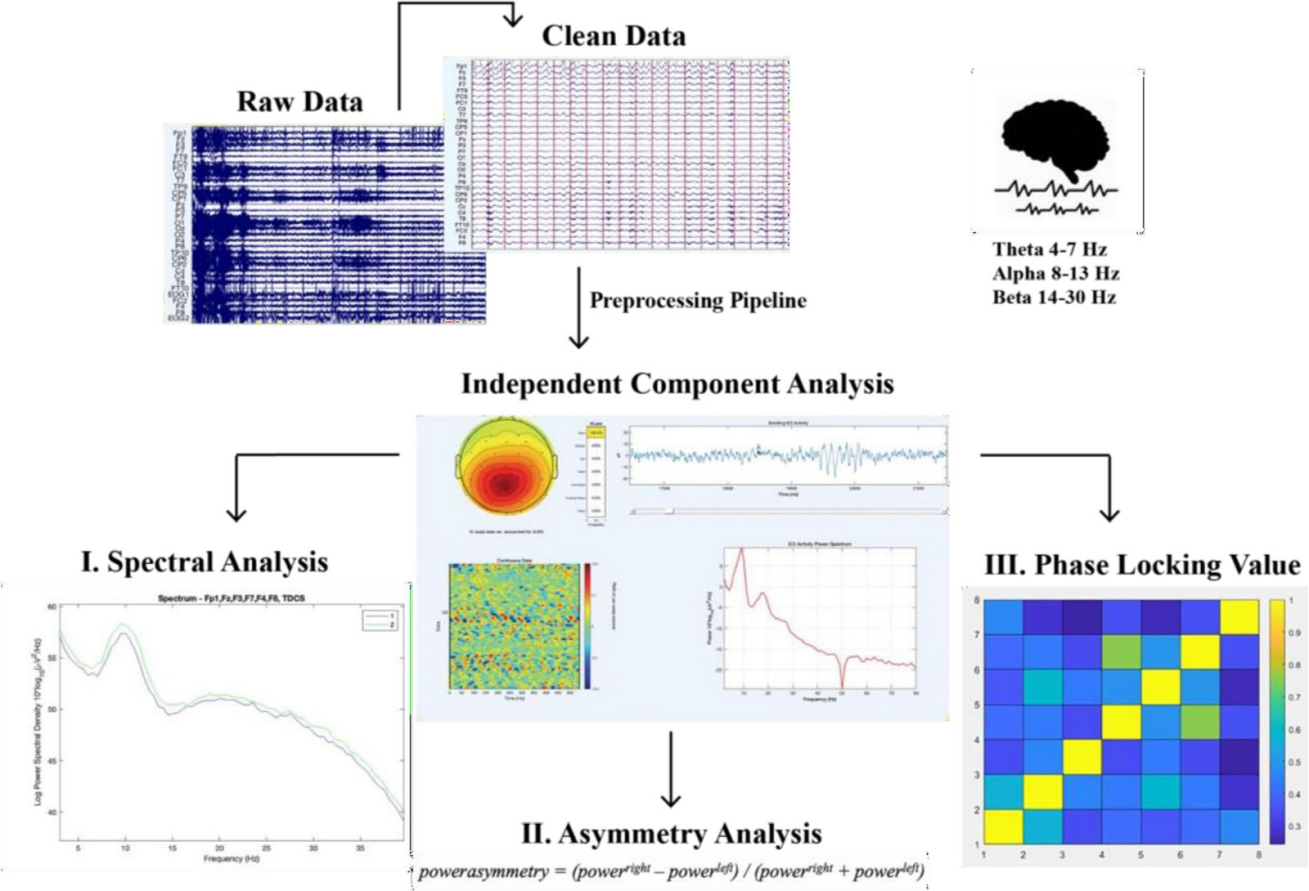

For an overview of the described analysis see Fig. 2.

### Experimental procedure

For the experimental procedure of the study see the protocol paper (Sergiou et al., 2020a) and for an overview see Supplementary Materials S2.

### Statistical analyses

Statistics were performed using the SPSS 25.0 software (Statistical Package for Social Sciences, SPSS Inc, Chicago). We used three analyses of variance for repeated measures (rm-ANOVA) to examine if there was a difference between active HD-tDCS and the sham condition for our main region of interest (i.e., the prefrontal cortex). Data are reported as means and standard deviations. Mauchly's test was used to test for sphericity, and the Greenhouse–Geisser correction was applied if necessary. A *p-*value of < 0.05 was considered significant.

A three-way repeated-measures ANOVA was used to test for the effect of condition (active HD-tDCS vs. sham) x lead (Fp1, Fz, F3, F4, F7, F8, FC1, FC2) x frequency band (theta, alpha, beta) calculated in rsEEG absolute power (μV^2^).

For the calculation of the asymmetrical activity three repeated measures ANOVA for each frequency band (theta, alpha, beta) were conducted to test whether this activity changed from pre to posttest for the two groups (HD-tDCS vs. Sham).

To compute phase synchronization, we extracted the PLV using the method described by Lachaux et al. (1999) and calculated the difference in symmetry between the electrode pairs that are part of the frontal region (Fz, Fp1, F3, F4, F7, F8, FC1, FC2) using a customized MATLAB script. We measured the connectivity between the electrodes using the Phase Locking Value (PLV). We conducted separate repeated-measures ANOVAs for the PLV of each chosen frontal electrode pair over Time (pretest vs posttest) with group (HD-tDCS vs Sham) as a between subjects factor.

## Results

Overall, neither side effects nor any relevant discomfort was reported during or after the HD- tDCS intervention (see Sergiou et al., 2022). Also, no difference in medication use was found between the two groups (tDCS and sham) X2 (3, N = 50) = 3.1, *p* = .378 (see S4).

Furthermore, there was no difference in substance use between the two groups X2 (2, N = 50) = 2.0, *p* = .368 (See S5).

### Effect of HD-tDCS on the frequency power

The results of the analysis on the absolute EEG power revealed significant main effects of lead (F (1.79,73.7) = 1.09, *p* < .01, *η ^2^* = 0.34), and frequency band (F (1.31,53.7) = 22.7, *p* <.01, *η ^2^* = 0.36). In addition, we found a significant interaction effect for lead x frequency band ^(F (1.64,67.8) =20.9,^ *^p^* ^< .01,^ *^η^*p*^2^* ^= 0.34)^. Because we did not find any significant results for time or condition, we did not further investigate these effects.

### Effect of HD-tDCS on the asymmetrical frontal activity

Regarding the asymmetrical activity we did not find a significant effect for the theta or alpha frequency bands. For the asymmetrical activity in the beta frequency, we did not find a significant main effect for Time, but we did find a significant interaction of Time vs. Group F(1,41 =^4.39,^ *^p^* ^= .042,^ *^η 2^* ^= 0.09)^. An exploratory post-hoc analysis revealed that the effect was significant between groups at the pretest. When we examined this in more detail, we found that for a large subgroup in the HD-tDCS condition (*n* = 11) the baseline values were starting at negative (indicating a greater left-sided activity) and that for this subgroup the increase was significant (*p* < .001) towards a left to right sided cortical activity from pre- to post intervention.

### Effect of HD-tDCS on the connectivity (PLV)

**Fig. 3:**
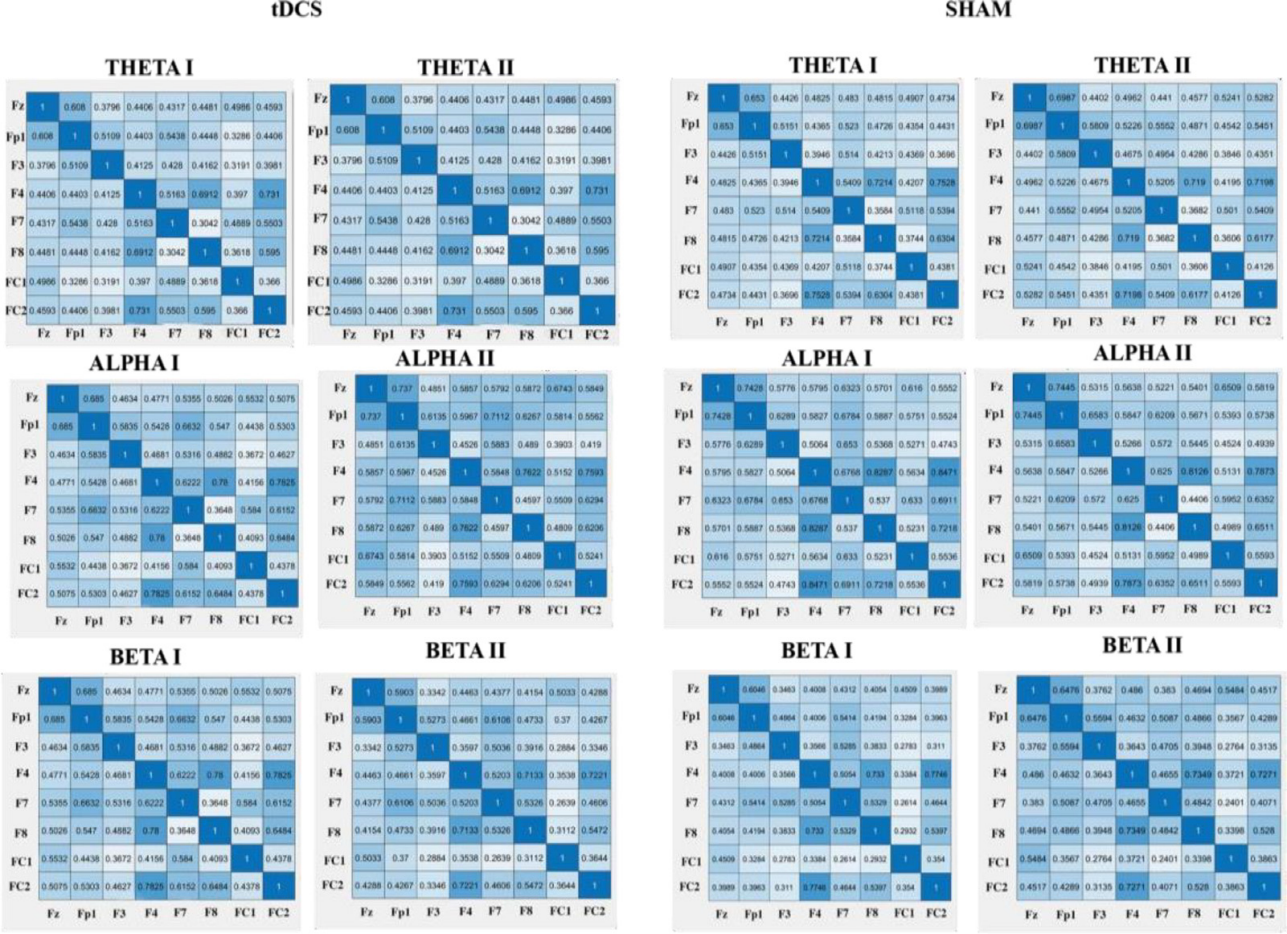

Regarding the effects of HD-tDCS on the PLV's, we did not find any significant results for the theta frequency band. However, for the alpha frequency band, we found a significant interaction for FP1-FC1 alpha (*F* (1,41) = 5.67, *p =* .022, *η 2* = 0.12) in HD-tDCS vs. sham in pre- vs. poststimulation. The HD-tDCS group showed an increase of connectivity as reported by higher PLV values from pretest (*M* = 0.44, *SD* = 0.26) to posttest (*M* = 0.58, *SD* = 0.19). In addition, we found a significant interaction for F7-FC1 alpha (*F* (1,41) = 6.15, *p =* .017, *ηp2* = 0.13). The HD-tDCS group showed an increase of connectivity as reported by higher PLV values from pretest (*M* =36, *SD* = 0.22) to posttest (*M* = 0.46, *SD* = 0.21). Regarding the beta frequency band, we found a significant interaction for F7-F8 beta (*F* (1,41) = 4.294, *p =* .045, *η 2* = 0.10) with an increase for the HD-tDCS group from pretest (*M* = 0.49, *SD* = 0.13) to posttest (*M* = 53, *SD* = 0.12) and a decrease for the sham group from pretest (*M* = 0.53, *SD* = 0.12) to posttest (*M* = 0.48, *SD* = 0.12). For an overview of the PLV values see Fig. 3.

## Discussion

Using HD-tDCS targeting the vmPFC, we investigated the effect of brain modulation on neural oscillations during rsEEG in a sample of violent forensic patients with a substance dependence. The aim of the current study was threefold.

First, we studied the effects of modulation on frontal power in the theta, alpha, and beta frequency band. Contrary to our predictions, we did not find an effect of the HD-tDCS modulation on the frequency power in these frontal areas. This finding may be explained in several ways. First, although the importance of focusing on frontal regions of the brain in violent offenders has emerged from multiple studies (i.e., Carvor & Harmon-Jones, 2009; Harmon-Jones, 2003; Hofman & Schutter, 2009, 2013; Peterson et al., 2008; Schutter et al., 2008), it could be that the modulation in spectral power was more prominent in other regions of the brain. For example, in a recent study of Konicar et al. (2021) they found excessive slow-wave activity limited to temporal and parietal regions instead of frontal, this could explain we did not find an effect of the intervention in the theta frequency band from pre- to posttest. Future studies should investigate a whole brain analysis to examine in which brain region the effect of HD-tDCS on power might be detected.

Our second aim was to investigate the effects of modulation on frontal asymmetrical activity in different frequency bands. For the theta frequency band, we did not find an effect of the HD-tDCS modulation on asymmetrical activity. This finding is not surprising, since the theta frequency band is mostly used to investigate cognitive motor functions (Mizelle et al., 2010) and our study is not a cognitive motor function study. Also, we did not to find an effect of modulation on asymmetrical activity in the alpha frequency band, which contradicts our hypothesis. However, closer inspection of the literature shows that this is not uncommon. For example, our null finding is in line with the findings reported in Hofman & Schutter (2012). In their study, they did not find any evidence for an association between HD-tDCS and frontal alpha asymmetrical activity and explained this by the assumption that alpha oscillations tend to be dominant over the posterior scalp locations during resting-state EEG recording. Therefore, a posterior-to-anterior direction of the configuration of the posterior dipole could explain the absent association between the alpha oscillations and frontal excitability (Hofman & Schutter, 2012) and this could also explain our lack of evidence in the current study. Another explanation could be that the association between asymmetrical activity in forensic patients may also be dependent on individual characteristics in personality (Knyazev et al., 2008), which is not taken into account in the present study. For example, in the study of Savostyanov et al. (2009) the synchronization of electrophysiological cortical activity posttest was modulated by trait anxiety Another reason for not finding an association between alpha asymmetry and aggressive behavior is comparison with a previous studie that did (Keune et al., 2012), might be that in that study the focus was more in including types of aggression (e.g., reactive vs. proactive), existence of psychopathic traits and/or comorbidity, and that these elements might explained why they did find results and we did not.

Last, it might be that the timing of the day and year of when the patients received the modulation could be of influence. Harmon-Jones et al. (2010) predicted that the baseline levels of asymmetrical frontal cortical activity correlate with basal cortisol levels and that relative right frontal activity was indeed greatest mornings in fall . Our study was carried out in summer and had one morning measure and one afternoon measure. In future studies, we should take the timing of asymmetrical activity into account. Therefore, more research is needed to better understand state variance in resting-state EEG and how this can be optimized in future studies.

For the beta frequency band, we observed an interaction between asymmetrical activity and group at the pretest, but we did not find increased asymmetrical activity using the HD-tDCS intervention over time for the complete HD-tDCS group. The results partly confirm our hypothesis, and are partly consistent with previous studies (Hoffman & Schutter, 2012; Schutter et al., 2008; Schutter & Hofman, 2009). Although we did not find the results for the effect over time, exploratory analysis revealed an increase in a small group of the HD-tDCS condition (see 3.2), with a negative (indicating a greater left-sided activity) baseline level and that for this subgroup the increase was significant towards a left to right sided cortical activity. Although no conclusions can be drawn here, it would be interesting to further investigate whether the effectiveness of the modulation depends on the baseline activity.. . Interestingly, our findings are also in line with previous research suggesting that more left than right-sided activity may interact with interventions in terms of positive treatment outcomes (Smith et al., 2017). Therefore, it could be that the group with more left-sided activity did respond to the HD-tDCS in terms of altering cortical activity and did show no results for the other participants.

Furthermore, it could be that we found different results in the power frequency bands and asymmetrical activation because our forensic patients had a history of drug dependence and were in abstinence. A recent systematic review (Liu et al., 2022) has demonstrated that alcohol/substance use disorders (AUD/SUDs) are associated with abnormalities in the rsEEG and that a larger part of these abnormalities are not spontaneously recovered after short abstinence. In addition, the timing of the intervention in the stage of the treatment as usual could be of crucial importance. Each patient that participated in our study was in a different stage of their treatment in the forensic clinic; some of them were already there for a long time, some just arrived, and others were just there for a transfer period in between clinics. Hence, it could be that those individual differences in the stage of their treatment contributed to the effectiveness of HD-tDCS in modulating the rsEEG. For example, Volkow et al. (1993) demonstrated that during the first week of abstinence, an elevated cerebral metabolism in the PFC has been found that explained the decreased activity in the PFC after the first week, due to lower brain dopamine. Meaning that metabolic changes seen in substance users during detoxification are related to changes in dopamine activity, thereby accounting for differences in the effects of tDCS. Therefore, the impact of HD-tDCS on altering synaptic plasticity could be of importance in the different stages of neurocognitive recovery (Verveer et al., 2021). Finally, our third aim was to investigate the effect of HD-tDCS modulation on resting-state connectivity (expressed as PLV) in frontal brain regions. Our finding of an increase in resting- state connectivity from pre- to posttest in the alpha and beta frequency band is consistent with our hypothesis. More specifically, we found a significant increase in alpha connectivity in the left hemisphere for the HD-tDCS group compared to the sham group. Again, here we find that the left hemisphere is better responding to the HD-tDCS treatment. Former studies linked the increase of alpha-band connectivity to enhanced cognitive demand (Pijnenburg et al., 2004; Palva et al., 2010; Wianda and Ross, 2019) in healthy and Alzheimer patients. . EEG coherence represents the covariance between two electrode locations in their spectral activity and can therefore be considered as a rough measure in temporal synchronicity between neural populations (Babiloni et al., 2006). Higher connectivity can be seen as increased functionality in controlling cognitive abilities such as impulsivity, response time or executive functioning (Machida et al., 2019). Therefore, it could be promising to use methods (such as HD-tDCS) to increase the connectivity, and thereby also functionality, within the brain of violent offenders. When enhancing synchronicity and activity in the frontal region it could lead to a alterations in the deficits present in the brain of violent offenders that might trigger aggressive behavior.

For the beta frequency band, we found a significant increase in functional connectivity between the left and right hemisphere in frontal areas. Since the brain relies on the interhemispheric transfer of information to integrate behavior and cognition (Doron and Gazzaniga, 2007), for the larger part in the axonal transmissions between the two hemispheres sub-served by the corpus callosum (Banich, 1998), this is a key element in functional connectivity and with that increased functioning of the brain. Thus, increased connectivity affects the transfer between the hemispheres and with that interhemispheric connectivity is reinforced to influence cognitive performance and improve the functioning of the frontal brain region. Studies support this with stating that a decreased connectivity is associated with cognitive impairement (Babiloni et al., 2006; Keary et al., 2009; Kumar et al., 2009; Thiruvady et al., 2007). It may be implicated that increased connectivity within the alpha and beta frequency band found in the current study may potentially result in alterations in the processing of moral emotions, emotion regulation and violent behavior. That is, the frontal cortex includes various regions that are involved in emotion regulation and emotional expressed aggression, violence as well as moral action and judgement (e.g., Harmon-Jones, 2004; Rosell and Siever, 2015). A large body of literature demonstrates that synchronized synaptic excitation is much more likely to evoke an action potential in neurons than (temporally) uncorrelated cues (Palva and Palva, 2011). In other words, synchronization thus provides a neuronal population an advantage in engaging the target neurons (Singer, 1999; Uhlhaas et al., 2009). With brain regions in greater coherence, thus syncing more in their neuronal oscillatory activity, the communication between brain regions can be enhanced. Therefore, it could be implicated that diminished brain activation in frontal brain regions in violent offenders with a diagnosis of substance dependency patients can be enhanced using tDCS.

Using HD-tDCS to increase neural connectivity can therefore be an interesting revenue for further studies on rehabilitation in forensic settings. The outcomes of this study could provide important knowledge about dysfunctions in neural oscillations associated with aggression in offenders, and the possible modulation of this neural activity in offenders using HD-tDCS technology (i.e. it has shown to influence specific neural mechanisms involved). Furthermore, it could contribute to the effectiveness of HD- tDCS as a supplementary treatment in forensic populations.

### Limitations

Some limitations on the generalizability and specificity of the present outcomes must be considered when interpreting the results. First, it must be acknowledged that the current sample consisted of violent male forensic patients with substance dependence, and therefore we were not able to draw any conclusions about neural oscillations related to aggression in offenders more generally, nor for those offenders who are violent and are diagnosed with an antisocial personality disorder. Future studies have to be conducted in other samples to support generalizability of our results. Importantly, although current findings were the result of experimentally induced changes using HD-tDCS targeting the vmPFC, current findings were not compared to data from a healthy control group. Future studies should include these (control) groups to accurately determine whether the results found are specific to violent forensic patients with a substance dependence, or might me more generally related to offender populations. In addition, for future studies it would be very interesting to link asymmetrical activity to aggression outcomes, since multiple studies demonstrate the association between asymmetrical frontal activity and aggressive tendencies (Hofman & Schutter 2009, 2012; Schutter et al., 2008). In that matter, it could enhance our understanding of the aggressive behavior of forensic patients and how underlying cortical activity plays a role in this.

Second, as shown in earlier studies (Carvor & Harmon-Jones, 2009; Hofman & Schutter, 2012; Peterson et al., 2008; Schutter et al., 2008) the characteristics of the EEG recording in the localization of cortical activity sources and the claims made in this study regarding the activity over the brain regions should be interpreted with caution to ensure the effects originates from the cortical tissue beneath the electrode positions.

Third, in this study, resting-state EEG data during eyes closed was used, as is similar to the approach used in other studies (Jacobson et al., 2012a; Keeser et al., 2011b). However, it could be that data from the eyes open condition might have resulted in other outcomes. Other studies on resting-state EEG have used data collected when participants had their eyes open, to prevent the possibility that participants were asleep during the eyes closed condition. (Boonstra et al., 2016) and those studies did find changes in mean frequency in rsEEG after tDCS. This methodological difference may have affected our outcomes, as the aftereffects of tDCS have previously been shown to depend on the physiological state during tDCS administration (Antal et al., 2007) and therefore our choice for analyzing only data from the eyes closed condition might have influenced our results.

Fourth, it should be noted that aggression is a very heterogeneous construct (Rosell & Siever, 2015) which might result in heterogeneous variability in brain activation between different type of violent offenders. Although no studies are reporting the same effect for alpha or beta in antisocial individuals, it might be that these frequency bands also have a more pronounced effect located in other brain regions. Future studies should investigate whether these aberrant brain processes, characteristically for antisocial or violent individuals, are linked to specific regions because this could provide important treatment implications. In addition, investigating a whole-brain analysis should be a logical follow-up to this study to see whether the effects were present in other brain regions in our sample.

One of the main takeaway notes is to investigate the brain regions that are characteristic of the forensic sample tested. Results can vary based on electrode locations (frontal, parietal, temporal, etc.) and the methods used to calculate connectivity. For example, we used the PLV, but recent studies also indicate the use of the Phase Lag Index (PLI) as a value measurement in calculation synchronicity. The choice of all these parameters contributes to a better understanding of modulating electrophysiological measures and the possible modulation of neural activity with HD-tDCS leading towards an implementation in treatment.

### Conclusion

The present study was the first to investigate modulation of neural dynamics using HD-tDCS in rsEEG in a sample of violent forensic patients with a substance dependency analyzing three different indices of neural dynamics. In our results, we found no evidence for a modulation effect of HD-tDCS on spectral power in the theta, alpha and beta frequency bands. However, we did find increased left-frontal asymmetrical activity after HD-tDCS in the beta frequency band, but this was only in a subsample of the forensic patients.

Furthermore, we find increased connectivity in frontal regions in the alpha and beta frequency bands as a result of HD-tDCS modulation, indicating enhanced synchronicity between frontal regions.. This study has enhanced our understanding of the neural underpinnings of aggression and violence, pointing to the importance of alpha and beta frequency bands and their connectivity in frontal brain regions. Although future studies should further investigate the complex neural underpinnings of aggression in different populations, it can be suggested with caution, that HD-tDCS could be an innovative method to regain frontal synchronicity in neurorehabilitation.

## Appendix Supplementary materials

Image, application 1

## Footnotes

Supplementary material associated with this article can be found in the online version at doi:10.1016/j.ijchp.2023.100374.
