# Supplementary material for: The effect of HD-tDCS on brain oscillations and frontal synchronicity during resting-state EEG in violent offenders with a substance dependence
Source: Int J Clin Health Psychol. 2023 Feb 21;23(3):100374. doi: 10.1016/j.ijchp.2023.100374 (PMC9982047; doi:10.1016/j.ijchp.2023.100374)
Supplement: Supplementary file 1 [file mmc1.docx]

**The effect of HD-tDCS on brain oscillations and frontal synchronicity during resting-state EEG in violent substance dependent offenders.**

**Supplement**

**Supplemental Methods**

**S1.Figure S1.** CONSORT (Consolidated Standards of Reporting Trials) of the screening and enrollment of the participants who were randomly assigned to the active vmPFC-tDCS condition or the sham condition.

**
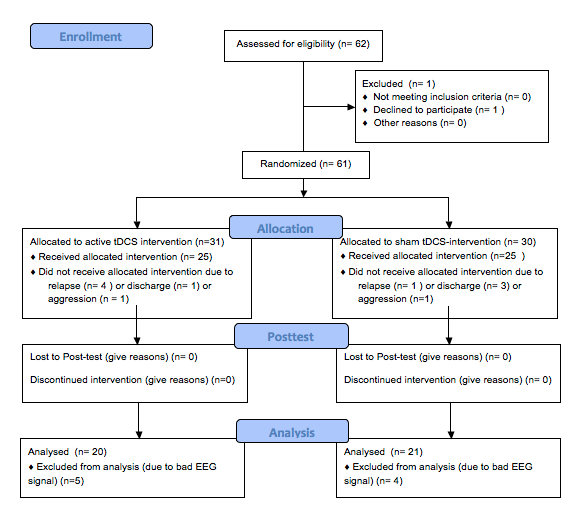
**

**S2. Figure S2.** Experimental procedure.

**
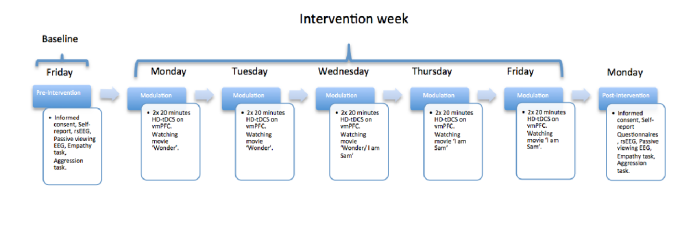
**

*Note*: EEG= Electroencephalography, rsEEG= Resting-state Electroencephalography, HD-tDCS= High-Definition transcranial Direct Current Stimulation, vmPFC= Ventromedial Prefrontal cortex.

**S3. Biophysical modeling and HD-tDCS simulation**

The induced E-field of the HD-tDCS montage was computed in SimNIBS v3.2, an open-source simulation package that integrates segmentation of MRI scans, mesh generation, and FEM E-field computations (Windhoff et al., 2013). The software provides a realistic volume conductor head model, created as default in FEM model generated using the T1-and T2-weighted images and segmentation from the SimNIBS example data set. The data sample was acquired from a healthy subject within the approval of the Ethics Committee of the Medical Faculty of the University of Tübingen (Windhoff et al., 2013). The data corresponds to a healthy subject (Ernie), which includes white matter, gray matter, cerebrospinal fluid, bone, and scalp tissue volumes. In our simulation we kept the isotropic conductivities given by default (Thielscher et al., 2015). The final mesh, comprehensive of gray and white matter, scalp, bone, and cerebrospinal fluid, comprises approximately 200,000 nodes and 3.6 million tetrahedral elements (see Windhoff et al., 2013 for further modeling details).

E-field distribution was computed within the SimNIBS model. We modeled the electrodes starting from the set used in the experiment, as small cylinders, 6 x 1cm radius (π cm²),with homogeneous conductivity and added to a 0.5mm in height corresponding approximately to electrode gel. The placement of the electrodes followed the 32 standard 10-20 Electroencephalography (EEG) system on the scalp with anodal high definition transcranial direct current stimulation (HD-tDCS) with electrodes over the Fpz (2mA) and cathodal tDCS over AF3, AF4, F3, F4 and Fz (-0.4mA each). The resulting Norm E-field and Normal E-field distribution is shown in Gmsh v4.7.1 (Geuzaine & Remacle, 2006) with an output range from 0 to 0.25 V/m.

We chose the measure of NormE in order to test the absolute intensity of the E-field induced, but this only give us an information of the strength of the electric field as vector, and therefore irrespective of the direction. Because we are interested in activating the vmPFC thanks to the anodal current in FPz, we also computed the NormalE of the E-field (see Figure 1, panel C). The resulting map represents the excitatory stimulation in ventral prefrontal areas, opposed to the cathodal stimulation spreading over the dorsal frontal and parietal regions.

**S4. Table S1. Medication use not associated with difference in groups (tDCS and Sham).**

tDCS (*N*=25) Sham (*N*=25)

n (%) n (%)

Non* 21 (84) 17 (68)

SSRI 2 (8) 2 (8)

Stimulants 2 (8) 4 (16)

Both 0 (0) 2 (8)

**Note.* Non= No SSRI or Stimulants.

Chi-Squaretest demonstrated that there was no significant relationship between medication and condition (3, N = 50) = 3.1, p = .378.

**S5. Table S2. SUD/AUD not associated with difference in groups (tDCS and Sham)**

tDCS (N=25) Sham (N=25)

n (%) n (%)

SUD 15 (60) 13 (52)
AUD 1 (4) 4 (16)
SUD+AUD 9 (36) 8 (32)

Chi-Squaretest demonstrated that there was no significant relationship between substance use and condition X2 (2, N = 50) = 2.0, p = .368

**Supplemental References**

Geuzaine, C., & Remacle, J.-F., 2008. Gmsh reference manual: the documentation for Gmsh,
 a finite element mesh generator with built-in pre-and post-processing facilities. URL
 http://www. geuz. org/gmsh.

Thielscher, A., Antunes, A., Saturnino, G.B., 2015. Field modeling for transcranial magnetic
 stimulation: A useful tool to understand the physiological effects of TMS? Annu Int
 Conf IEEE Eng Med Biol Soc 2015, 222–225.
 https://doi.org/10.1109/EMBC.2015.7318340.

Windhoff, M., Opitz, A., Thielscher, A., 2013. Electric field calculations in brain stimulation
 based on finite elements: An optimized processing pipeline for the generation and
 usage of accurate individual head models. Human Brain Mapping 34, 923–935.
 https://doi.org/10.1002/hbm.21479.
